# Deficiency of histone deacetylases 3 in macrophage alleviates monosodium urate crystals-induced gouty inflammation in mice

**DOI:** 10.1186/s13075-024-03335-4

**Published:** 2024-05-06

**Authors:** Qi-Bin Yang, Meng-Yun Zhang, Liu Yang, Jie Wang, Qing-Sheng Mi, Jing-Guo Zhou

**Affiliations:** 1 Department of Rheumatology and Immunology, Affiliated Hospital of North Sichuan Medical College, Nanchong, Sichuan Province 637000 People’s Republic of China; 2 Department of Integrated TCM and Western Medicine, General Hospital of Central Theater, PLA, Wuhan, Hubei Province 430070 China; 3 Henry Ford Immunology Program, Departments of Dermatology and Internal Medicine, Henry Ford Health System, 1 Ford Place, Detroit, MI 48202 USA; 4 Department of Rheumatology and Immunology, Clinical Medical College, The First Affiliated Hospital of Chengdu Medical College, Chengdu, Sichuan Province 610500 People’s Republic of China

**Keywords:** Gout, HDAC3, Macrophage polarization, HDAC3 inhibitor, Monosodium urate crystals

## Abstract

**Background:**

Gout is caused by monosodium urate (MSU) crystals deposition to trigger immune response. A recent study suggested that inhibition of Class I Histone deacetylases (HDACs) can significantly reduce MSU crystals-induced inflammation. However, which one of HDACs members in response to MSU crystals was still unknown. Here, we investigated the roles of HDAC3 in MSU crystals-induced gouty inflammation.

**Methods:**

Macrophage specific HDAC3 knockout (KO) mice were used to investigate inflammatory profiles of gout in mouse models in vivo, including ankle arthritis, foot pad arthritis and subcutaneous air pouch model. In the in vitro experiments, bone marrow-derived macrophages (BMDMs) from mice were treated with MSU crystals to assess cytokines, potential target gene and protein.

**Results:**

Deficiency of HDAC3 in macrophage not only reduced MSU-induced foot pad and ankle joint swelling but also decreased neutrophils trafficking and IL-1β release in air pouch models. In addition, the levels of inflammatory genes related to TLR2/4/NF-κB/IL-6/STAT3 signaling pathway were significantly decreased in BMDMs from HDAC3 KO mice after MSU treatment. Moreover, RGFP966, selective inhibitor of HDAC3, inhibited IL-6 and TNF-α production in BMDMs treated with MSU crystals. Besides, HDAC3 deficiency shifted gene expression from pro-inflammatory macrophage (M1) to anti-inflammatory macrophage (M2) in BMDMs after MSU challenge.

**Conclusions:**

Deficiency of HDAC3 in macrophage alleviates MSU crystals-induced gouty inflammation through inhibition of TLR2/4 driven IL-6/STAT3 signaling pathway, suggesting that HDAC3 could contribute to a potential therapeutic target of gout.

**Supplementary Information:**

The online version contains supplementary material available at 10.1186/s13075-024-03335-4.

## Introduction

Gout is caused by monosodium urate (MSU) crystals deposition and characterized by recurrent self-limiting attacks of acute arthritis [1]. MSU crystals were viewed as a “danger signal” that could be recognized by Toll-like receptors (TLR) 2/4 to trigger the myeloid differentiation factor 88 (MyD88) pathway that led to nuclear factor κB (NF-κB) activation resulting in interleukin (IL)-1β release [2–4]. IL-1β could further amplify NF-κB signaling pathway to give rise to secondary inflammatory cascade, such as production of chemokines, IL-6, tumor necrosis factor (TNF)-α and so on [5–7]. MSU crystals-induced the classic pro-inflammatory response was occurred in macrophages, indicating that macrophage played a key role in gouty inflammation.

Histone deacetylase (HDAC), which was an enzyme to regulate gene expression via deacetylation of histone, could fold up chromatin to inhibit the genes expression, while Histone acetyltransferase (HAT) could unfold DNA to make genes transcribed [8]. A previous study revealed that inhibition of Class I HDAC could significantly reduce MSU crystals-induced cytokine production [9]. However, which one of 4 members of class I HDACs (HDAC1, HDAC2, HDAC3, HDAC8) that played a critical role in MSU crystal-induced gouty inflammation was still unknown. It was reported that HDAC3 was responsible for almost inflammatory genes expression in macrophage stimulated with lipopolysaccharide [10]. Furthermore, HDAC3 was described as a critical molecule in the deacetylation of NF-κB p65 which was required for inflammatory response [11]. Therefore, it was hypothesis that HDAC3 played a key role in MSU crystal-induced gouty inflammation.

In this study, macrophage specific HDAC3 knockout mice were used to explore the roles of HDAC3 in MSU crystals-induced gouty inflammation. We evaluated diverse types of murine gout models containing foot pad arthritis, ankle arthritis and subcutaneous air pouch *in vivo.* In addition, bone marrow-derived macrophages (BMDMs) were performed to clarify the mechanisms of HDAC3 in gouty inflammation in vitro.

## Methods

### Mice

The floxed HDAC3 was described previously [12]. Briefly, C57BL/6J mice expressing Cre recombinase gene under the control of the colony stimulating factor 1 receptor (Csf1r) promoter were bred to HDAC3^fl/fl^ to generate macrophage specific HDAC3 knockout (KO) mice (pairs of littermate mice, defining Csf1rCre^+^HDAC3^fl/fl^ as HDAC3 KO, Csf1rCre^−^HDAC3^fl/fl^ as HDAC3 WT) [13]. 8–12 weeks-old both age and gender-matched mice were applied to perform experiments. Handling of mice and experimental protocols were in accordance with the guidelines of the Institutional Animal Care and Use Committee (Henry Ford Health System).

### Gout models

***Air pouch model*** Mice were placed under anesthesia and injected with 5 mL of sterile air subcutaneously into the back of mice to form an air pouch, 3 mL air was injected into the air pouch on days 3 and 5. On day 7, MSU crystals suspension (3 mg of in 1mL PBS) were injected into the air pouch [14]. After MSU crystals injection at indicated time points (3, 6–12 h), mice were sacrificed and then air pouch cavity was washed with 2 mL PBS. Supernatants of air pouch lavage fluids (APLF) were collected for cytokines assessment and the cells from APLF were harvested for cellular phenotype analysis.

**Footpad and ankle joint model** Mice were anesthetized and then injected with MSU crystals suspension into footpad (1 mg in 40 µL PBS) or ankle joint (0.5 mg in 20 µL PBS) [14]. The size of paw swelling as well as ankle swelling were measured with an electronic caliper at the indicated time points (0, 3, 6, 12, 24, 48 and 72 h).

### BMDMs culture and treatment

Bone marrow cells obtained from HDAC3 KO or WT mice were cultured in Iscove’s Modification of Dulbecco’s Modified eagle Medium (IMDM) (#10-016-CV, Cellgro) with 10% fetal bovine serum (FBS) (#SV30014.03, HyClone), penicillin (100 units/mL) and streptomycin (100 µg/mL). To induce proliferation and differentiation of bone marrow cells to macrophages, the medium was supplemented with 30 ng/mL macrophage colony-stimulating factor (M-CSF) (#0914245, Peprotech). On day 7, the harvested BMDMs were treated with MSU crystals (50 µg in 1 mL RPMI-1640 ) for indicated time points. According to the experimental protocol for the in vitro experiment, the BMDMs were primed with 50ng/mL lipopolysaccharide (LPS) for 4 h before the BMDMs stimulated with MSU crystals suspension for 4–8 h. In the in vitro experiment of HDAC3 inhibitor, BMDMs were pretreated with dimethyl sulfoxide (DMSO) or 1 𝜇M RGFP966 (Selleckchem) for 24 h before MSU crystals suspension treatment [15, 16]. In macrophage polarization experiment, The BMDMs were pretreated with 10 ng/mL IL-4 or vehicle (bovine serum albumin) for 24 h [12].

### Flow cytometry analysis

Single-cell suspension was incubated with Fc block (clone 2.4G2) for 15 min and then the cells were stained with conjugated monoclonal antibodies, including Ly-6G (1A8), F4/80 (BM8), CD11b (M1/70) and TNF-α (MP6-XT22). All antibodies were purchased from eBioscience. Data were acquired by CellQuest software (BD Biosciences) and analyzed by FlowJo software (Tree Star Inc).

### Real-time quantitative polymerase chain reaction

Total RNA from BMDMs were extracted with Mammalian Total RNA Miniprep Kit (Sigma, USA) according to the manufacturer’s protocols. Real-time quantitative polymerase chain reaction (qPCR) was performed using the Quant Studio 7 Flex Real-Time PCR System (Applied Biosystems, USA). Relative gene expressions were normalized with glyceraldehyde-3-phosphate dehydrogenase (GAPDH) and analyzed by 2^*−ΔΔCT*^ method. The gene primers sequences were synthesized by Eurofins Genomics (Louisville, USA) and listed in Supplementary Table 1.

### Enzyme-linked immunosorbent assay (ELISA) analysis

TNF-α (#88-7321), IL-1β (#88-7013), IL-6 (#88-7064) in supernatants were assessed using ELISA Ready-Set-Go kit from eBioscience (San Diego, CA) following the manufacturer’s instructions. The 96-well microplates were read utilizing a VICTOR X3 Multilabel Plate Reader (PerkinElmer, USA).

### Western blot analysis

Proteins in BMDMs were extracted by RIPA lysis buffer (Thermo Scientific) containing protease or phosphatase inhibitors. The proteins (50–70 µg) were separated by 10% SDS-PAGE and transferred to the polyvinylidene fluoride (PVDF) membrane (Bio-Rad). The PVDF membrane was blocked in 5% nonfat milk (Sigma, USA) for 1 h at room temperature and then incubated with primary antibodies [anti-HDAC3 antibody (#85,057), anti-STAT3 antibody (#9139), anti-p-STAT3 antibody (#9131), and anti-β-actin antibody (#3700) (all antibodies from Cell Signaling Technology, dilutions 1:1000)] at 4℃ overnight. The secondary antibodies conjugated to horseradish peroxidase were incubated for 1 h at room temperature. Immunoreactive proteins were visualized using enhanced chemiluminescence system (Amersham Biosciences), according to the manufacturer’s instructions.

### Statistical analysis

Data were performed with Graphpad Prism Software (V.5.0f). Data are presented as means ± SD. Differences between experimental groups were analyzed using the unpaired-T test (two-tailed). *P* values less than 0.05 was considered statistically significant (*P < 0.05, **P < 0.01, ***P < 0.001).

## Results

### Deletion of HDAC3 in macrophages in mice

**Fig. 1:**
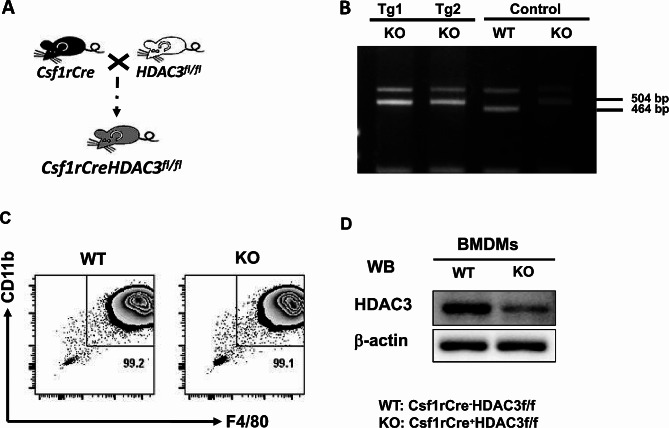
Generation of deletion of a loxP-flanked HDAC3 target gene in Csf1rCre mice. **A** C57BL/6J mice expressing Cre-recombinase expressed under the control of the Colony stimulating factor 1 receptor (Csf1r) promoter were mated with HDAC3f/f mice to generate macrophage specific HDAC3 knockout (KO) mice. **B** Genotyping of transgenic and conditional HDAC3 deletion mice. Resulting PCR products on agarose gel. DNA are extracted from the tail of target mouse and labeled as Tg1 and Tg2. **C** The BMDMs were represented by positive F4/80 and CD11b and the purity of BMDMs was identified with flow cytometry. **D** Confirmation of HDAC3 KO in macrophage by western blot. WT: Csf1rCre^−^HDAC3f/f. KO: Csf1rCre^+^HDAC3f/f

As shown in Fig. 1A, the C57BL/6J mice expressing Cre recombinase gene under the control of the colony stimulating factor 1 receptor (Csf1r) promoter were mated with HDAC3^fl/fl^ mice to generate macrophage specific HDAC3 knockout (KO) mice (Csf1rCre^+^HDAC3^fl/fl^). Littermates carrying HDAC3^fl/fl^ without cre expression (Csf1rCre^−^HDAC3^fl/fl^) were used as wild-type (WT) controls. Mice with transgenic HDAC3 deletion were identified by genotyping (Fig. 1B). The conditional HDAC3 KO in macrophage was confirmed in BMDMs using cellular phenotype. The purity of BMDMs was comparable between HDAC3 KO and WT mice (Fig. 1C). In addition, protein level of HDAC3 was significantly reduced in HDAC3 KO mice compared to WT mice (Fig. 1D).

### HDAC3 deficiency alleviated MSU crystals-induced footpad edema and ankle swelling in mice

**Fig. 2:**
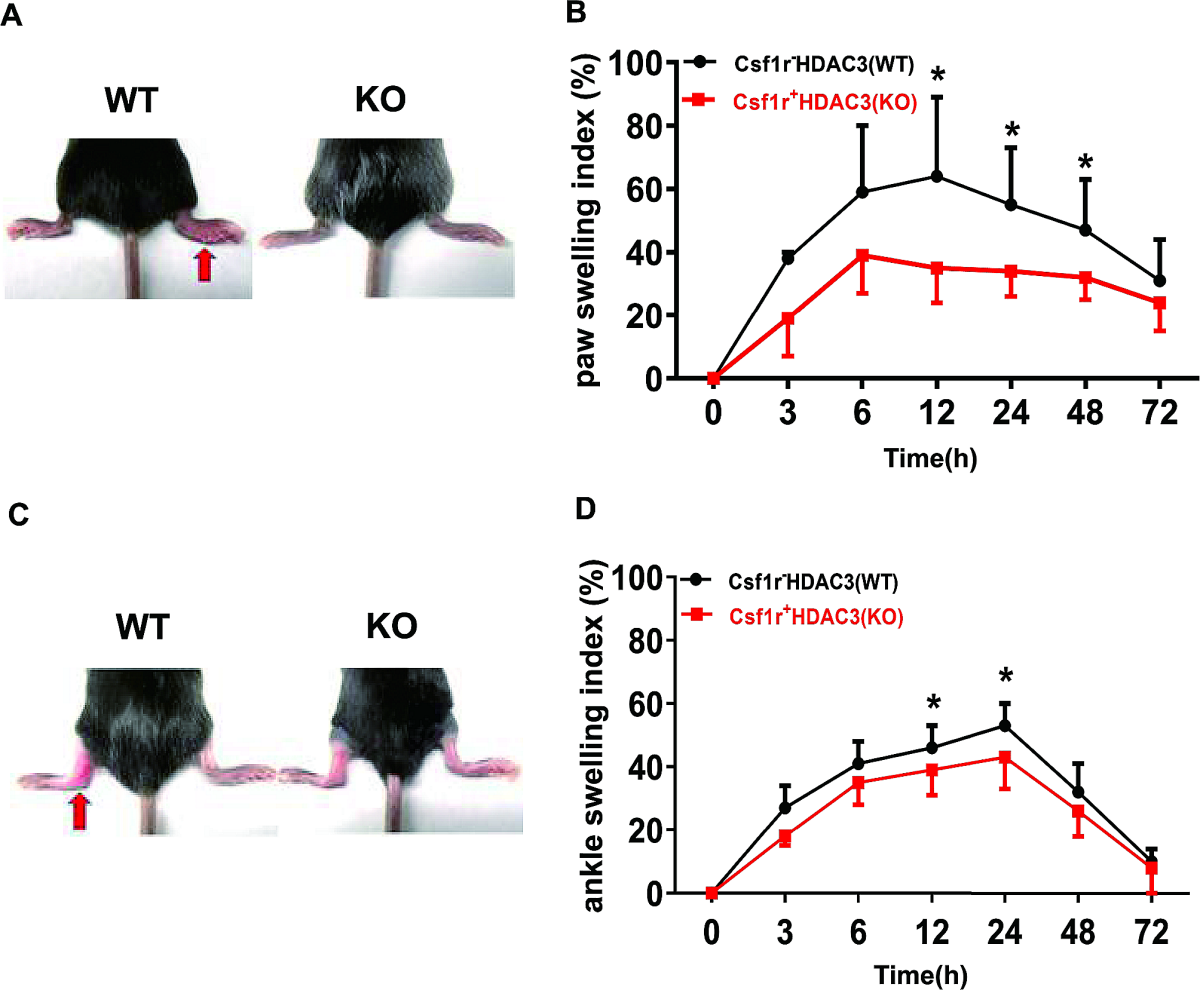
HDAC3 deficiency alleviated MSU crystals-induced footpad edema and ankle swelling in mice. **A, B** Changes in hind paw thickness and representative photographs of hind paws at 24 h time point after injection of MSU into contralateral footpads of Csf1r-HDAC3 (WT) mice and Csf1r + HDAC3 (KO) mice. **C, D** Changes in ankle joint thickness and representative photographs of ankle joint at 24 h time point after injection of MSU into contralateral ankle joint of Csf1r-HDAC3 (WT) mice and Csf1r + HDAC3 (KO) mice. Data were shown as mean ± SD from three independent experiments (*n* ≥ 9 mice per group). (*P < 0.05, by unpaired two-tailed Student’s t test)

To explore the clinical phenotype effect of HDAC3 in gout, the footpad and ankle joint models were used to mimic acute human gouty arthritis. After injection of MSU crystals into footpad for 12, 24 and 48 h, paw swelling index was significant decreased in HDAC3 KO mice compared to WT controls (Fig. 2A and B). Consistent with decreased paw swelling index, HDAC3 KO mice displayed less severe ankle joint swelling after injection of MSU for 12 and 24 h (Fig. 2C and D). These results indicated that HDAC3 deficiency alleviated MSU crystals-induced footpad edema and ankle swelling in mice, suggesting the anti-inflammatory effect of HDAC3 deficiency in macrophages during the development of acute gouty arthritis in mice.

### HDAC3 deficiency inhibited MSU crystals-induced inflammatory responses in air pouch model

**Fig. 3:**
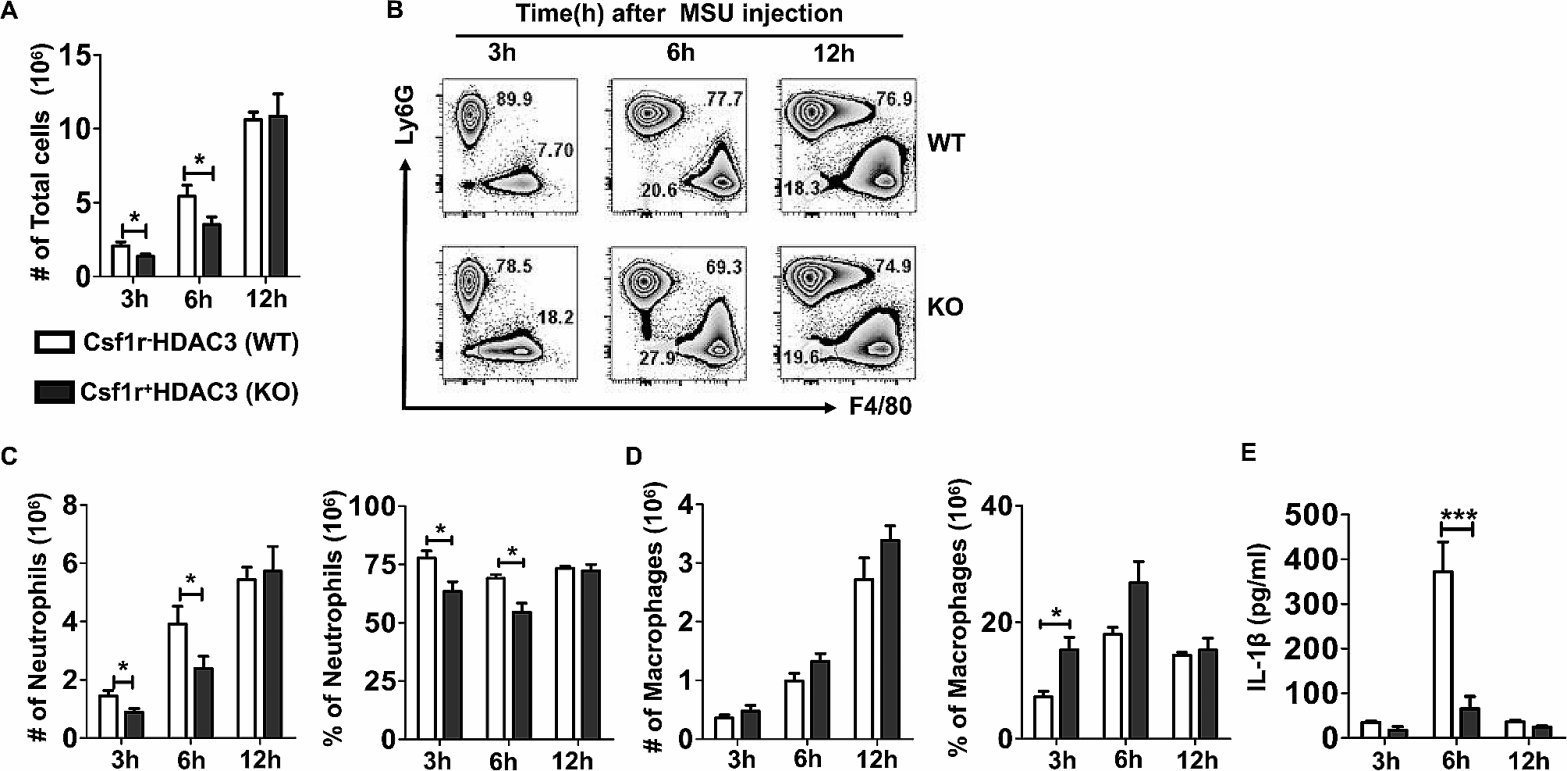
HDAC3 deficiency inhibited MSU crystals-induced inflammatory responses in air pouch model. **A** Number of total cells in air pouch lavage fluids (APLF) from WT and KO mice after MSU injection at indicated time points. **B** Flow cytometry analysis of APLF from WT mice and KO mice. Neutrophils were represented by Ly6G^+^, while macrophages were represented by F4/80^+^. **C, D** Number and ratio of neutrophils (**C**) and macrophages (**D**) in APLF from WT and KO mice after MSU injection at indicated time points. **E** Level of IL-1β in APLF supernatant was measured by ELISA. Data were shown as mean ± SD from three independent experiments (*n* ≥ 9 mice per group). (*P < 0.05, ***P < 0.001, by unpaired two-tailed Student’s t test)

To further evaluate the function of HDAC3 in the process of gouty inflammation, we performed the synovial like air pouch model with MSU crystals treatment at different time points. As shown in Fig. 3A, in comparison with WT mice, the total number of infiltrating cells into air pouch cavity of HDAC3 KO mice was significantly reduced at 3–6 h after MSU crystals challenge. Furtherly, the inflammatory cells neutrophils (Ly6G^+^) and macrophages (F4/80^+^) were identified by flow cytometry. We observed that the absolute number and frequencies of neutrophils were dramatically decreased in HDAC3 KO mice at 3–6 h (Fig. 3C). Even though the increased number of macrophages did not have a significant difference, the frequencies of macrophages in HDAC3 KO mice were significantly increased compared with WT mice after injection of MSU for 3 h (Fig. 3D). These data from air pouch model indicated that deletion of HDAC3 inhibited MSU crystals-induced gouty inflammation through inhibition of neutrophils recruitment into inflammatory site.

To shed more light on the effect of HDAC3 deficiency on cytokines responses during the onset of gouty inflammation, we also assessed cytokines production in air pouch cavity model. In comparison with WT mice, the secretion of IL-1β was dramatically decreased in HDAC3 KO mice after injection of MSU for 6 h (Fig. 3E). Considering IL-1β has been established as a pivotal cytokine involved in promoting a self-perpetuating inflammatory cascades, it suggested that HDAC3 deficiency could reduce MSU crystals-induced inflammatory responses via reduction of IL-1β release to amplify the inflammatory cascade.

### HDAC3 deficiency inhibited MSU crystals-induced TNF-α production in BMDMs

**Fig. 4:**
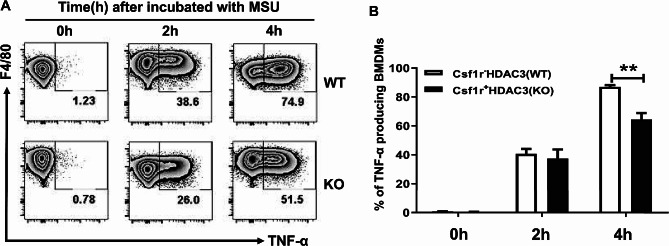
HDAC3 deficiency inhibited MSU crystals-induced TNF-α production in BMDMs. **A** Flow cytometry analysis of TNF-α producing BMDMs (represented by F4/80^+^TNF-α^+^) from WT and KO mice. **B** Percentage of TNF-α producing BMDMs from WT and KO mice at the indicated time points. Data were shown as mean ± SD from three independent experiments (*n* = 9 mice per group). (**P < 0.01, by unpaired two-tailed Student’s *t* test)

MSU crystals-induced acute inflammatory response are frequently driven by well-defined transcription factors, particularly, the nuclear factor (NF)-κB p65. NF-κB p65 activation is required for many kinds of pro-inflammatory cytokines such as tumor necrosis factor (TNF)-α [17]. It was reported that HDAC3, as a specific member of the HDAC family, was capable of regulating TNF-α production [18]. Since the HDAC3 KO mice has a favorable phenotype of reduced acute gouty inflammatory response in vivo, the in vitro functional experiment was performed to assess the level of pro-inflammatory gene TNF-α in BMDMs. In comparison with WT mice, the percentage of BMDMs producing TNF-α was remarkably decreased in HDAC3 KO mice with MSU crystals treatment at 4 h (Fig. 4A and B), although it was comparable at 2 h. Therefore, the results further verified that deletion of HDAC3 in macrophages played an anti-inflammatory role in MSU crystals-induced gouty inflammation.

### HDAC3 deficiency suppressed MSU crystals-induced genes related to TLR2/4 driven IL-6/STAT3 pathway in BMDMs

**Fig. 5:**
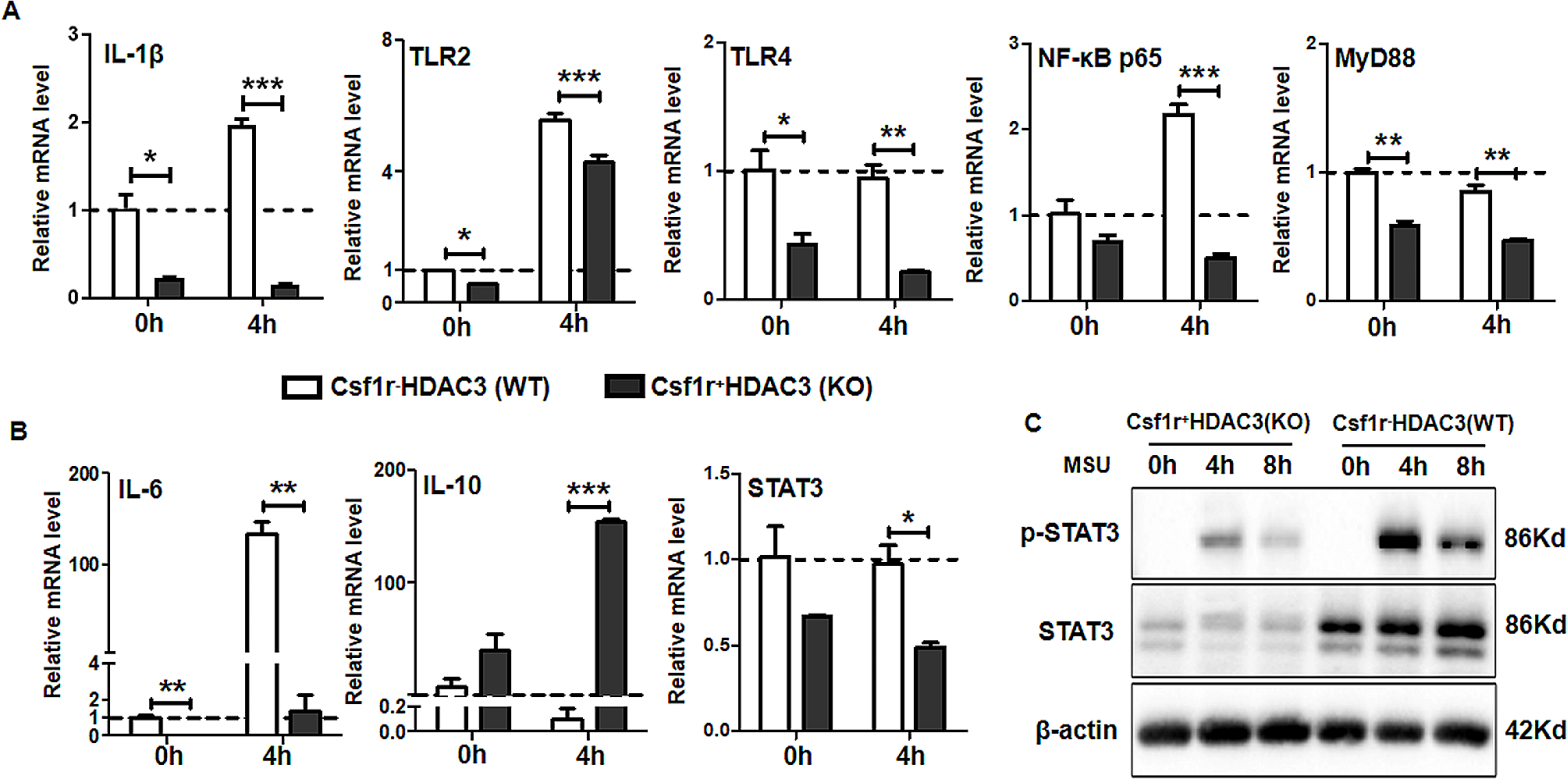
HDAC3 deficiency suppressed MSU crystals-induced genes related to TLR2/4 driven IL-6/STAT3 pathway in BMDMs. **A, B** Relative mRNA expression of IL-1β, TLR2, TLR4, NF-κB p65, MyD88, IL-6, IL-10 and STAT3 were measured in BMDMs from WT and KO mice incubated with 50ng (20µL, 2.5 mg/mL) MSU crystals for indicated time points. Relative gene expressions were normalized to GAPDH as fold changes versus control group (WT-MSU-0 h). **C** STAT3 and p-STAT3 protein level were analyzed in BMDMs from WT and KO mice incubated with 50ng (20µL, 2.5 mg/mL) MSU crystals for indicated time points. Data were shown as mean ± SD from three independent experiments (*n* = 9 mice per group). (*P < 0.05, ** *P* < 0.01, *** *P* < 0.001, by unpaired two-tailed Student’s *t* test)

To determine the mechanism of HDAC3 implicated in gouty inflammation, the genes related to TLR2/4 driven IL-6/STAT3 pathway were investigated. We found that mRNA level of the genes related to TLRs signaling pathway, such as TLR2, TLR4, MyD88, NF-κB p65 and IL-1β, significantly decreased in HDAC3 KO mice (Fig. 5A). Furthermore, treatment with MSU crystals for 4 h, the mRNA level of IL-6 and STAT3 decreased remarkably whereas IL-10 increased in BMDMs from HDAC3 KO mice (Fig. 5B). The HDAC3 deficiency inhibited STAT3-activated inflammation through increasing IL-10 and decreasing IL-6 production, indicating HDAC3 deficiency had a potential role to shift pro-inflammatory response toward an anti-inflammatory response. To further assess the effect of HDAC3 deficiency on STAT3 phosphorylation, we detected the levels of STAT3 and phosphorylated STAT3 (p-STAT3) protein in BMDMs treated with MSU crystals for 4–8 h. The protein level of STAT3 and p-STAT3 in HDAC3 KO mice were remarkably decreased (Fig. 5C), which was in accordance with the decreased mRNA level of STAT3. Over all, these data demonstrated that HDAC3 deficiency had an impressive anti-inflammatory effect on TLR2/4 driven IL-6/STAT3 pathway in MSU crystals-induced gouty inflammation in vitro.

### Specific HDAC3 inhibitor decreased MSU crystals-induced TNF-α and IL-6 production in BMDMs

**Fig. 6:**
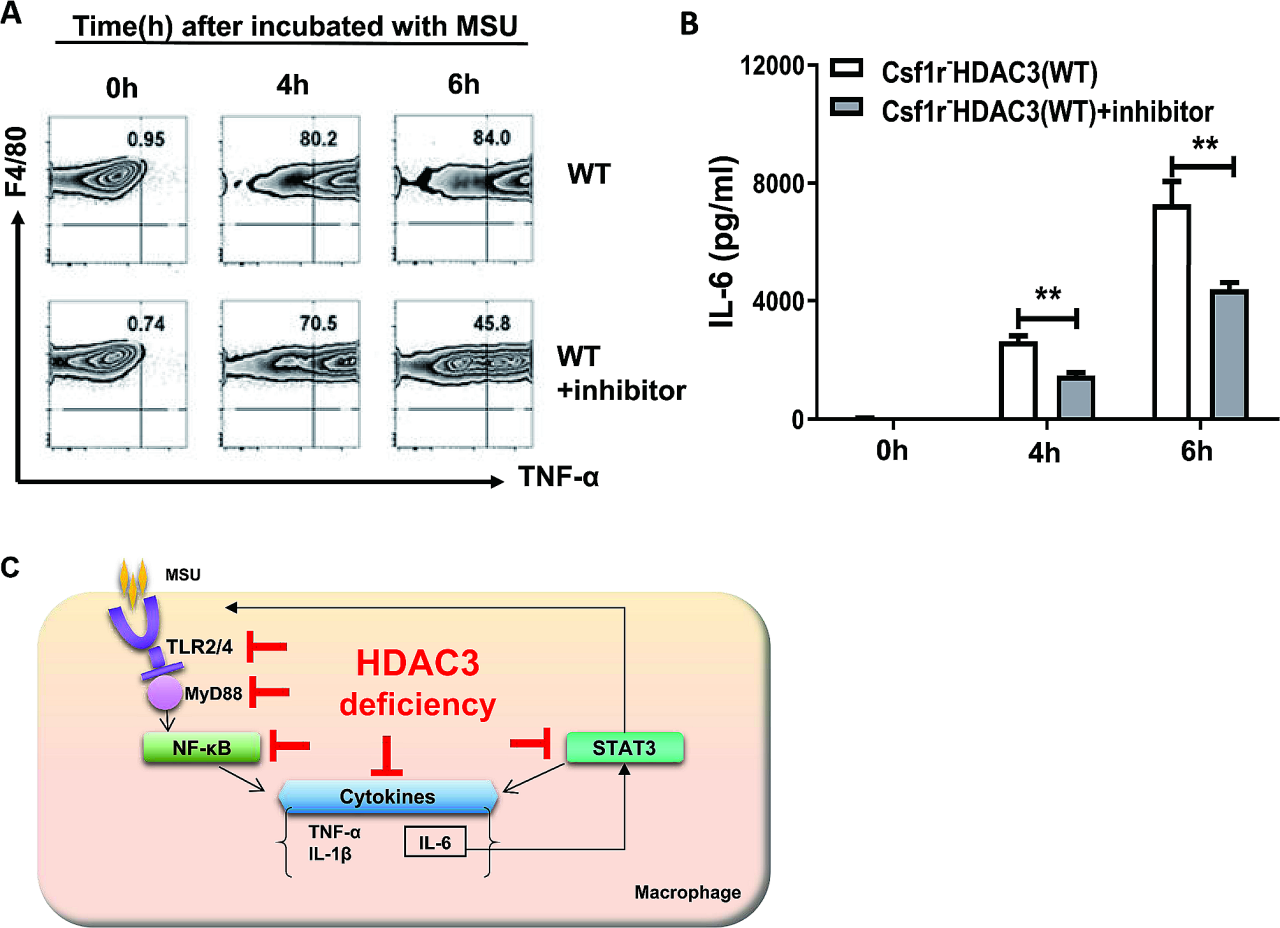
Specific HDAC3 inhibitor decreased MSU crystals-induced TNF-α and IL-6 production in BMDMs. **A** The TNF-α producing BMDMs (represented by F4/80^+^TNF-α^+^) were analysed in WT group and WT treated with HDAC3 inhibitor group (RGFP966). **B** The Levels of IL-6 were measured by ELISA in supernatant of BMDMs culture medium from WT group and WT treated with HDAC3 inhibitor group (RGFP966). **C** The schematic diagram showed that HDAC3 deficiency in macrophages inhibited TLR2/4, MyD88, NF-κB, STAT3 and pro-inflammatory cytokines production. Data were shown as mean ± SD from three independent experiments (*n* = 12 mice per group). (**P < 0.01, by unpaired two-tailed Student’s *t* test)

It was evidenced that macrophage-specific HDAC3 deficiency had an anti-inflammatory role in MSU crystals-induced gouty inflammation both in vivo and in vitro. To further explore the potential clinical therapeutic effect of HDAC3 inhibitor, RGFP966 was investigated. RGFP966 was a competitive tight-binding HDAC3 inhibitor, with an IC50 of 0.08µM for HDAC3 and without effective inhibition of any other HDAC at concentrations up to 15µM [15]. Firstly, 1µM of RGFP966 as the optimum concentration was confirmed in BMDMs from WT mice upon MSU crystals (Data not shown). Secondly, the BMDMs from HDAC3 WT mice were pre-treated with 1µM of RGFP966 for 24 h, and subsequently incubated with MSU for 4–6 h. We found that the percentage of BMDMs producing TNF-α was significantly decreased compared with controls (Fig. 6A). Moreover, in comparison with controls, the levels of IL-6 in the supernatant of RGFP966 inhibitor group were obviously decreased (Fig. 6B). Those data demonstrated that RGFP966, the specific HDAC3 inhibitor, could decrease MSU-induced inflammatory cytokines production. Taken together, based on the findings from specific HDAC3 inhibitor and HDAC3 KO mice, the mechanism of macrophage special HDAC3 deficiency which decreased pro-inflammatory cytokines production in MSU crystals-induced gouty inflammation could attribute to suppression of genes related to TLR2/4 driven IL-6/STAT3 pathway, such as TLR2/4, MyD88, NF-κB, IL-1β, IL-6, TNF-α and STAT3 (Fig. 6C).

### HDAC3 deficiency shifted gene expression from pro-inflammatory macrophage (M1) to anti-inflammatory macrophage (M2) in BMDMs

**Fig. 7:**
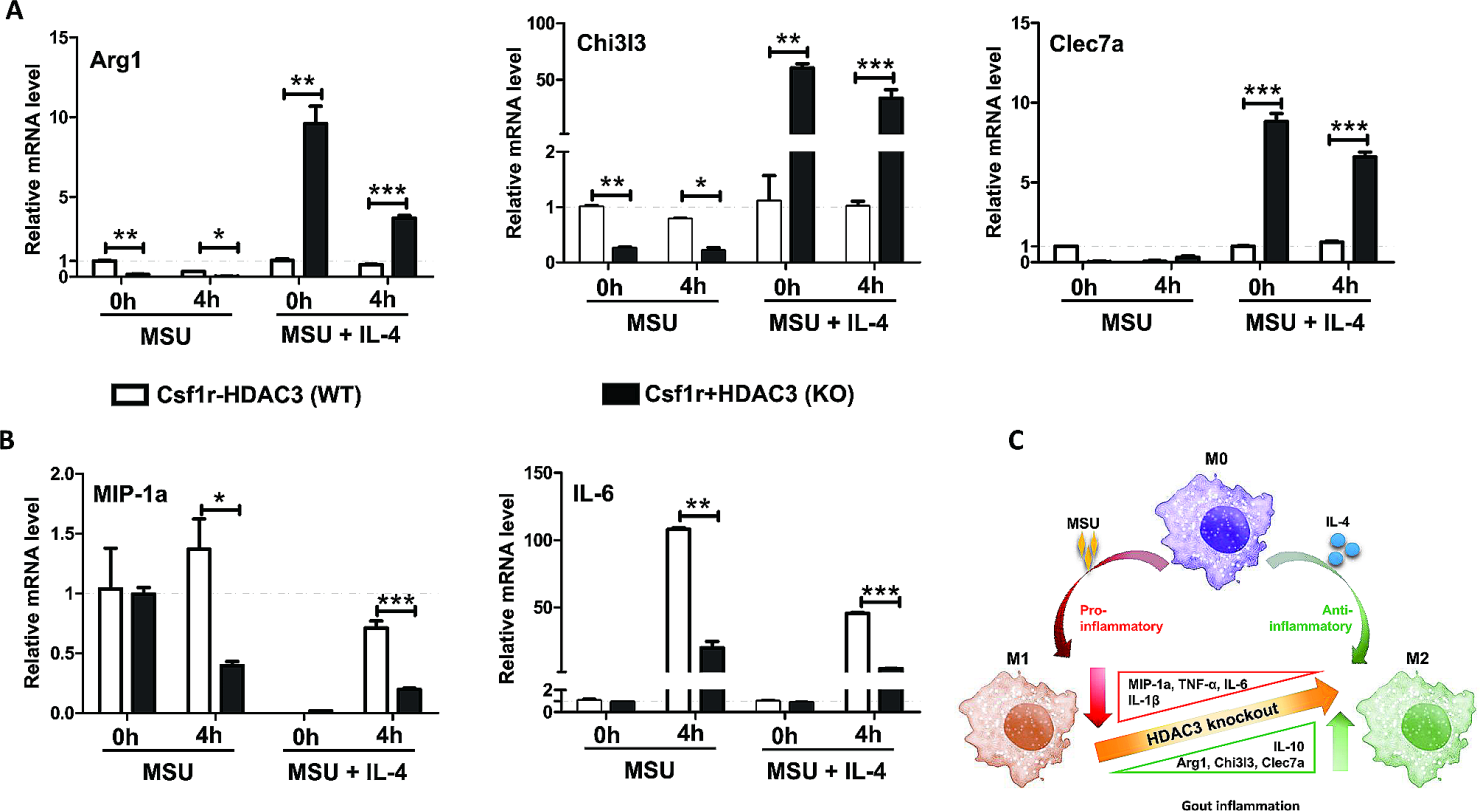
HDAC3 deficiency shifted gene expression from pro-inflammatory macrophage (M1) to anti-inflammatory macrophage (M2) in BMDMs. **A, B** Relative mRNA expression of Arg1, Chi3l3, Clec7a, MIP-1a and IL-6 were measured in BMDMs from WT and KO mice incubated with 50ng (20µL, 2.5 mg/mL) MSU crystals for indicated time points. Relative mRNA expressions were normalized to GAPDH as fold changes versus control group (WT-MSU-0 h). **C** Increased M2 marker gene and decreased M1 marker gene were observed in macrophage HDAC3 deficiency, indicating a shift from pro-inflammatory to anti-inflammatory macrophage. Data were shown as mean ± SD from three independent experiments (*n* = 9 mice per group). (*P < 0.05, **P < 0.01, ***P < 0.001, by unpaired two-tailed Student’s t test)

To assess the effect of HDAC3 deficiency on macrophage polarization upon MSU crystals-induced inflammation, the BMDMs were incubated with IL-4 for 24 h to induce macrophage differentiation into M2-like macrophages phenotype. The genes of pro-inflammatory M1 macrophages and anti-inflammatory M2 macrophages in response to MSU crystals-induced inflammation were investigated. It was observed that the expression of M2 marker genes, including Arg1, Chi3l3 and Clec7a (Fig. 7A), were remarkably increased in BMDMs from HDAC3 KO mice with pre-treatment of IL-4 in contrast to those without pre-treatment of IL-4. However, the M1 marker genes, including MIP-1a and IL-6 (Fig. 7B), were significantly decreased upon MSU stimulation. HDAC3 deficiency suppressed M1 macrophages polarization in response to MSU stimulation and promoted a shift from M1 to M2 macrophages in the presence of IL-4 (Fig. 7C), suggesting the shift from pro-inflammatory to anti-inflammatory macrophage could be one of remission mechanisms of gouty inflammation.

## Discussion

HDAC3 has been identified as a crucial epigenetic regulator of inflammatory response in several inflammatory diseases [19–23], Considering the emerging role of macrophages is highlighted as having a critical role in both the initiation and resolution of acute gout [24], it is significant and imperative to study on the role of macrophage-specific HDAC3 deficiency in gouty inflammation to investigate the mechanism of gout development and explore available therapies. In the current study, we made mouse model with conditional deletion of HDAC3 in macrophage to investigate the roles of HDAC3 in development and function of MSU-induced inflammation. We showed here that deficiency of HDAC3 in macrophage alleviates MSU crystals-induced gouty inflammation via inhibition of TLR2/4 driven IL-6/STAT3 signaling pathway and also promote a functional macrophage shift from pro-inflammation to anti-inflammation.

We firstly investigated the influence of HDAC3 deficiency on MSU crystals-induced inflammation in gout mouse models in vivo. It was observed that HDAC3 KO mice had a favorable phenotype to reduce acute gouty arthritis since macrophage-specific HDAC3 deficiency in MSU crystals-induced footpad edema and ankle swelling models (Fig. 2). Furtherly, less severe infiltration of neutrophils and attenuation of pro-inflammatory cytokines IL-1β production in response to MSU stimulation were the major features in air pouch model (Fig. 3). These results were consistent with a previous study that reported HDAC3-deficient macrophages could almost lack half of inflammatory genes expression [10], suggesting that HDAC3 deficiency played an anti-inflammatory role in the development of gout.

Considering TLR2/4 and its related downstream signaling pathways involved in the inflammatory cascade of MSU crystals-induced gouty inflammation [2, 25]. Particularly, the downstream molecules including MyD88, NF-κB, IL-1β, IL-6, TNF-α [26, 27] and signal transducer and activator of transcription 3 (STAT3) [28, 29]. Since TLR2/4 has been identified as STAT3 target genes [30, 31] and IL-6 upregulated TLR2/4 expression via IL-6/STAT3 pathway [32, 33], a central role of NF-κB in a feedback loop that included TLR/NF-κB/IL-6/STAT3/TLR has been established [34]. Emerging evidence suggested that HDAC3 was involved in the deacetylation of the NF-κB in IL-1 signaling pathway which played a key role in gouty inflammation [11, 18]. Here, we demonstrated that HDAC3 deficiency inhibited the genes transcription related to TLR2/4 driven IL-6/STAT3 pathway, enhanced the transcription of IL-10 and downregulated the translation of TNF-α, STAT3 and p-STAT3 in BMDMs (Fig. 4). It was reported that MyD88 inhibition could block gouty inflammation in mouse models by down-regulating inflammatory signals, including NF-κB and autocrine IL-6/IL-10 engagement of the JAK-STAT3 pathway [27]. Inactivation of NF-κB signaling pathway exerted its anti-inflammatory effects by inhibiting TNF-α protein expression in gouty inflammation [26]. Besides, As a downstream effector of cytokine signal transduction pathways and a latent cytoplasmic transcription factor identified as an acute-phase response factor, STAT3 was essential in MSU-induced inflammatory response [28, 29]. Several studies have shed more light on that the abnormal activation of TLR2/4 signaling pathway and its related downstream pro-inflammatory cytokine production (such as IL-1 and IL-6) was mediated by STAT3 [31]. As an important downstream effecter of TLR signaling pathway, IL-6 not only enhanced TLRs induced inflammatory response via STAT3 activation [32] but also potentiated pro-inflammatory cytokine and chemokine production stimulated by TLRs [35, 36], indicating that a positive feedback existed between IL-6 production and the activation of TLR2/4 via STAT3 [30, 32, 37, 38]. In addition, IL-6 was associated with inflammatory activity in gout, the presence of tophi and articular deformities [39] and IL-10 down-regulated the inflammatory responses in gout through inhibition of TNF-α, MIP-1α, and MIP-1β in MSU crystal–stimulated cells [40]. Our results were supported that IL-10 induced prolonged activation of STAT3 led to an anti-inflammatory response whereas IL-6 activated STAT3 to induce acute pro-inflammatory response [41]. Taken together, our findings revealed that targeting genes related to TLR2/4 driven IL-6/STAT3 pathway could be helpful to inhibit gouty inflammation.

It was reported that HDAC3 inhibitor interfered with TLR signaling pathway, STAT3/5 pathway and pro-inflammatory cytokines including IL-1β, IL-6 and TNF-α [22]. In our study, we investigate the pro-inflammatory cytokines in MSU-stimulated BMDMs with the pre-treatment of RGFP966. RGFP966 decreased MSU-induced TNF-α and IL-6 production in BMDMs (Fig. 5A and B), which was in line with our findings that HDAC3 deficiency in macrophages played anti-inflammatory roles in MSU crystals-induced gouty inflammation. Although there has been lacking of studies about HDAC3 inhibitor to challenge with MSU crystals-induced gouty inflammation in vivo, the identification of the anti-inflammatory role of HDAC3 deficiency may provide valuable clues for the future applications of selective HDAC3 inhibitor in gout patients.

The previous studies showed that the differentiation of MSU crystals-recruited monocytes into a pro-inflammatory M1-like macrophages phenotype were primarily responsible for producing pro-inflammatory cytokines during the onset of MSU crystals-induced inflammation [42, 43]. While classically activated macrophage (M1) contributed to the inflammatory response, alternatively activated macrophage (M2) presented an anti-inflammatory phenotype [12]. Macrophage lacking HDAC3 displayed a polarization phenotype which was similar to IL-4-induced alternative activation [12]. To explore the effects of HDAC3 deficiency on macrophage polarization, which played a causal role in onset of gouty inflammation, IL-4 was used to induce M2-like macrophage phenotype. In BMDMs from HDAC3 KO mice, the anti-inflammatory M2 macrophage marker genes (Arg1, Chi3l3 and Clec7a) were up-regulated with pre-treatment of IL-4 (Fig. 6A). In contrast, the pro-inflammatory M1 macrophage marker genes (MIP-1a and IL-6) were down-regulated upon MSU stimulation (Fig. 6B). Our findings were consistent with the study of HDAC3 inhibitor on inflammatory gene expression in lung tissue obtained up-regulation of anti-inflammatory M2 maker genes accompanied by down-regulation of pro-inflammatory M1 maker genes [21]. Notably, it seemed that down-regulation of M1 marker genes were only observed in BMDMs from HDAC3 KO mice after MSU-stimulation (Fig. 5B). It remained unknown to the mechanism of these results. Further studies were needed to prove the hypothesis that HDAC3 deficiency only affected macrophage polarization during the development of gouty inflammation and could not increase the expression of M2 marker genes without IL-4. Overall, it indicated that HDAC3 deficiency promoted a shift from pro-inflammatory to anti-inflammatory response in MSU crystals-induced gouty inflammation.

Collectively, our study provided evidence on the macrophage HDAC3 functions in animal model of gouty inflammation. Our research focused on macrophage-specific HDAC3 deficiency in development and function of MSU-induced inflammation via MSU-induced gout mouse models. Deficiency of HDAC3 in macrophage alleviated MSU crystals-induced gouty inflammation via inhibition of TLR2/4 driven IL-6/STAT3 signaling pathway and promoted macrophage shift from M1 to M2. Thus, here we offered insights that should be considered for the future researches on development of histone deacetylases in gout.

## Conclusion

Taken together all findings in this study, we concluded that macrophage-specific deficiency of HDAC3 prevented MSU crystals-induced gouty inflammation in mice. Conditional knockout of HDAC3 in macrophage did not only suppress MSU crystals-induced inflammation by inhibition of TLR2/4 driven IL-6/STAT3 pathway but also promote a functional macrophage shift from pro-inflammation to anti-inflammation in response to MSU crystals. RGF966, as a newly discovered HDAC3-selective inhibitor, demonstrated impressive inhibitory effects on pro-inflammatory cytokines. Further studies on the application of HDAC3 inhibitor in vivo were expected to evidence that targeting HDAC3 could be one of promising treatment for gout.

### Electronic supplementary material

Below is the link to the electronic supplementary material.

Supplementary Material 1

Supplementary Material 2

## Data Availability

The datasets used and/or analyzed during the current study are available from the corresponding author on reasonable request.

## Abbreviations

MSU: monosodium urate
HDACs: histone deacetylases
KO: knockout
BMDMs: bone marrow-derived macrophages
TLR: Toll-like receptors
MyD88: myeloid differentiation factor 88
NF-κB: nuclear factor κB
IL: interleukin
TNF: tumor necrosis factor
HAT: histone acetyltransferase
Csf1r: colony stimulating factor 1 receptor
APLF: air pouch lavage fluids
FBS: fetal bovine serum
DMSO: dimethyl sulfoxide
CSF: macrophage colony-stimulating factor
PVDF: polyvinylidene fluoride
GAPDH: glyceraldehyde-3-phosphate dehydrogenase
ELISA: enzyme-linked immunosorbent assay
STAT3: signal transducer and activator of transcription 3
M1: classically activated macrophage
M2: alternatively activated macrophage
