# Supplementary material for: Deficiency of histone deacetylases 3 in macrophage alleviates monosodium urate crystals-induced gouty inflammation in mice
Source: Arthritis Res Ther. 2024 May 6;26:96. doi: 10.1186/s13075-024-03335-4 (PMC11071232; doi:10.1186/s13075-024-03335-4)
Supplement: Supplementary file 2 — Supplementary Material 2 [file 13075_2024_3335_MOESM2_ESM.docx]

| **Supplementary Table 1 Primer Sequences** | | | |
| --- | --- | --- | --- |
|  | **Gene** | **Sequence (5′→3′)** |  |
| **genotyping**  **primers** | *HDAC3 1133T* | CTC TGG CTT CTG CTA TGT CAATG | Floxed exon 7 and wild-type exon 7 of the HDAC3 |
|  | *HDAC3 1597B* | GGA CAC AGT CAT GAC CCG GTC | Allele with primer set 1133T/1597B |
|  | **Gene** | **Forward Primer** | **Reverse Primer** |
| **qPCR**  **primers** | *IL-6* | *TCCAGTTGCCTTCTTGGGAC* | *GTACTCCAGAAGACCAGAGG* |
|  | *IL-10* | *CGGGAAGACAATAACTG* | *CATTTCCGATAAGGCTTGG* |
|  | *STAT3* | *GGGCCATCCTAAGCACAAAG* | *GGTCTTGCCACTGATGTCCTT* |
|  | *IL-1β* | GGGCCTCAAAGGAAAGAATC | CTCTGCTTGTGAGGTGCTGA |
|  | *TLR2* | CCAAAGAGCTCGTAGCATCC | AGGGGCTTCACTTCTCTGCT |
|  | *TLR4* | CAAGAACATAGATCTGAGCTTCAACCC | GCTGTCCAATAGGGAAGCTTTCTAGAG |
|  | *NF-κB p65* | CGATGCGATTAGTTCTGGCTTCCT | TGTGTCTTGGTGGTATCTGTGCTT |
|  | *MyD88* | CGGAACTTTTCGATGCCTTT | TAGTTGCCGGATCATCTCCT |
|  | *Arg1* | CTCCAAGCCAAAGTCCTTAGAG | AGGAGCTGTCATTAGGGACATC |
|  | *Chi3l3* | GGCTCAAGGACAACAATTTAGG | ACTGTGGAAAAACCGTTGAACT |
|  | *Clec7a* | TCATTGAAAGCCAAACATCG | CCTGGGGAGCTGTATTTCTG |
|  | *MIP-1α* | CAAGTCTTCTCAGCGCCATA | GGAATCTTCCGGCTGTAGG |
